# Acute respiratory infections and its associated risk factors among children aged 6–59 months in Ghana: a multinomial regression analysis of the 2022 demographic and health survey

**DOI:** 10.3389/fpubh.2025.1518427

**Published:** 2025-06-16

**Authors:** Desmond Klu, Amidu Alhassan, Charity Akpene Dansu

**Affiliations:** ^1^Centre for Malaria Research, Institute of Health Research, University of Health and Allied Sciences, Ho, Ghana; ^2^Department of Adult Health, School of Nursing and Midwifery, College of Health and Allied Sciences, University of Cape Coast, Cape Coast, Ghana; ^3^Kotobabi Number Two Junior High School, Ghana Education Service, Accra, Ghana

**Keywords:** acute respiratory infections, child health, maternal factors, household predictors, Ghana

## Abstract

**Background:**

Acute Respiratory Infections (ARIs) remain a critical health concern, particularly among children aged 6–59 months, where they are among the leading causes of morbidity and mortality worldwide. Globally, ARIs significantly contribute to child mortality, accounting for nearly 15% of all deaths in children under 5 years of age.

**Objective:**

To assess the risk factors associated with ARI severity among children aged 6–59 months in Ghana.

**Research design:**

This study utilized data from the 2022 Ghana Demographic and Health Survey (GDHS), focusing on a weighted sample of 541 children aged 6–59 months who exhibited symptoms of ARI. Multinomial logistic regression analyses were conducted to examine maternal, household, and child-related predictors of ARI. A significant *p-value* was set at 0.05.

**Results:**

The prevalence of ARI was 17.1% for chest problems only, 66.1% for nose breathing difficulties, and 16.8% for both nose and chest problems. Key predictors of ARI included coastal zone residency (aOR = 7.89; CI: 2.26–27.60), maternal illiteracy (aOR = 0.34; CI: 0.12–0.93), maternal ethnicity (Akan: aOR = 0.12; CI: 0.02–0.85), and age of household head (20–29 years: aOR = 59.08; CI: 3.04–1,150.14). Boys were more likely than girls to experience both nose and chest problems (aOR = 3.44; CI: 1.61–7.34), and younger children, particularly those under 12 months, were at higher risk of ARI (aOR = 21.04; CI: 3.34–132.64). Children who were not breastfeeding (aOR = 2.62; CI: 1.15–5.94) and had not suffer from diarrhea (aOR = 2.28; CI: 1.19–4.38) were more at risk of ARI.

**Conclusion:**

The findings highlight the significant role of maternal education, household sanitation, and child-specific factors in influencing ARI risk among children in Ghana. Efforts to improve maternal education, enhance sanitation facilities, and implement targeted interventions for high-risk children are critical to reducing the burden of ARI in Ghana.

## 1 Introduction

Acute Respiratory Infections (ARIs) remain a critical health concern, especially among children aged 6–59 months, ranking as a leading cause of morbidity and mortality worldwide (1). Globally, ARIs account for nearly 15% of all deaths in children under 5 years of age (2), with over 800,000 children dying annually from ARIs, primarily due to pneumonia, the most severe form (2). In Africa, ARI prevalence among children under five who received antibiotics is particularly high in Tanzania (61%), Sao Tome and Principe (60%), Rwanda and Congo (58% each), Angola (56%), Mozambique (54%), Kenya (53%), Namibia (52%), and Gabon (50%) (3). In Ghana, 2% of children under age five exhibited symptoms of an ARI, while 15% had a fever, and 13% experienced diarrhea in the 2 weeks preceding the survey (4). A study by Kolekang et al. (5) suggested that scaling up basic health interventions, including immunization, exclusive breastfeeding, and improved sanitation could significantly reduce ARI-related mortality.

ARIs encompass infections of the upper and lower respiratory tracts, from mild conditions like the common cold to severe illnesses such as pneumonia and bronchiolitis. These infections are primarily caused by viral and bacterial, including *Streptococcus pneumoniae, Haemophilus influenzae type b* (Hib), respiratory syncytial virus (RSV), and influenza viruses (6). ARIs are highly contagious, transmitted through airborne droplets, direct contact, and contaminated surfaces, making them highly contagious. According to Sharrow et al. (7) ARIs are the most prevalent infectious diseases globally, and their impact on children is particularly severe due to their developing immune systems.

Children with ARIs typically present with symptoms such as coughing, difficulty breathing, fever, wheezing, and nasal congestion. In severe cases, children may develop chest in-drawing, rapid breathing, and lethargy, symptoms indicative of pneumonia, a leading cause of ARI-related deaths (8). Studies have shown that early recognition and treatment of these symptoms can reduce mortality. For instance, McAllister et al. (9) found that children who received timely medical interventions for ARIs had a 30% lower risk of severe complications.

Globally, ARIs are the second leading cause of death in children under five, with the highest burden in low- and middle-income countries (2). Africa and Southeast Asia experience 70% of global deaths from pneumonia and other respiratory infections (6). In developing countries, poor healthcare infrastructure, malnutrition, and environmental factors such as air pollution exacerbate ARI risks. According to WHO estimates, two-thirds of ARI-related deaths could be prevented through simple interventions like vaccinations, adequate nutrition, and access to clean water and sanitation (10).

In many developing countries, including Ghana, ARIs remain a major cause of death among children. High prevalence is driven by overcrowded living conditions, inadequate healthcare services, and widespread biomass fuels for cooking, which increases indoor air pollution (11). In Ghana, findings from the Demographic and Health Surveys (DHS) indicate that ARI symptoms among children aged 6–59 months decreased slightly between 2000 and 2014, but the rates remain high. Approximately 2% of children under age five showed ARI symptoms, 15% exhibited fever, and 13% experienced diarrhea in the 2 weeks preceding the survey (4).

In Ghana, ARI-related morbidity and mortality continue to pose public health challenges. ARI symptoms prevalence has slightly declined in recent years due to improved healthcare access and increased vaccination coverage (4), mortality rates from respiratory infections remain a concern, especially in rural areas with limited healthcare access. The use of antibiotics for ARIs in Ghanaian children has also increased, although there is growing concerns about antibiotic resistance due to inappropriate prescription practices (12).

Key risk factors associated with ARIs in children include malnutrition, low birth weight, and exposure to indoor air pollution from biomass fuels (4, 13). In developing countries particularly Ghana, children living in households using solid fuels for cooking are twice as likely to experience ARI symptoms compared to those in households using cleaner fuels (14). Socioeconomic determinants such as maternal education, wealth status, and access to healthcare services play critical roles. For instance, Makoka (15) found that children from wealthier households and those with more educated mothers were less likely to suffer from ARIs. Additionally, boys, younger children (under 2 years) and those in rural areas face higher ARI risks due to poor healthcare access and environmental factors like indoor air pollution (11).

Socioeconomic factors such as household wealth, maternal smoking, and vaccination status significantly affect ARI risk. In Ghana, children from poorer households are more likely to suffer from ARIs, especially where sanitation and access to clean water are inadequate (12). Poor sanitation, particularly in rural areas with limited access to improved toilet facilities, exacerbates the risk of respiratory infections (15). Despite extensive research on ARIs, there remains a gap in understanding the specific determinants of ARI severity and how various factors interact to influence health outcomes among children in Ghana. Previous studies have often focused on isolated risk factors without fully exploring the combined effects of environmental, socioeconomic, and behavioral determinants on ARI prevalence. This study uses multinomial regression analysis on 2022 Demographic and Health Survey data to assess the risk factors associated with ARIs among children aged 6–59 months in Ghana. By identifying the key determinants of ARI severity, this study seeks to inform targeted interventions to reduce child mortality and improve health outcomes in Ghana. Specifically, this study aligned with Sustainable Development Goal (SDG) target 3.2, which seeks to end preventable deaths of newborns and children under 5 years of age by 2,030.

## 2 Methods

### 2.1 Data source

Data for this study were obtained from the nationally representative 2022 Ghana Demographic and Health Survey (GDHS), conducted between October 17, 2022, and January 14, 2023. We used data from the children's file. The GDHS collects information on various topics, including child and infant mortality and morbidity, child nutrition, maternal health, household population and characteristics, and other demographic and health outcomes. In this study, data on a weighted subsample of children who had problems with their chest blocked nose, and difficulty breathing were extracted and analyzed.

### 2.2 Survey and study participants

Details concerning the scope and methodology of the GMIS have already been published^4^. The GDHS is a nationally representative survey conducted by the Ghana Statistical Service (GSS), Ministry of Health (MOH), and Ghana Health Service with technical support from the Inner-City Fund (ICF) through the Demographic and Health Surveys (DHS) Program. The data collection was performed in two phases. The first phase comprised the household listing exercise, during which each of the 200 selected enumeration areas was visited, and information was recorded on structures. Additionally, information on the names of household heads and the global positioning system (GPS) coordinates of clusters was collected. In the second phase, households and all eligible women (15–49 years) were interviewed, including mothers of children under age five.

### 2.3 Sampling and sample size

The 2022 GDHS included a total of 8,581 children aged 6–59 months. Out of this number, 1, 634 children were reported to have experienced a cough within the 2 weeks preceding the survey. However, for the purpose of this study, we focused on a subset of children who, not only had a recent cough but also exhibited symptoms such as chest-related problems, blocked or runny nose, and difficulty breathing, which are indicators suggestive of ARI. This sub-population comprised 541 children. To account for sampling design, including under-sampling and over-sampling, we applied a weighting adjustment using the DHS standard approach by dividing the weighting variable (v005) by 1,000,000 (v005/1,000,000). Therefore, the weighted sample of children aged 6–59 months with symptoms of acute respiratory infections in the 2022 GDHS was 541.

### 2.4 Outcome variable

The outcome variable for this study was children with acute respiratory infections (ARI). Children who were reported to show symptoms of ARI such as short, rapid breaths, difficulty in breathing, problems with the chest, or a blocked or runny nose were selected. To measure this variable, three questions were asked:

1. i. *Has (NAME) had an illness with a cough at any time in the last 2 weeks? Yes……………..[1]*
2. *No ………….[2]*
3. ii. *When (Name) had an illness with a cough, did he/she breathe faster than usual with short, rapid breaths or have difficulty breathing? Yes………………….[1]*
4. *No ………………[2]*
5. iii. *Was the fast or difficult breathing due to a problem in the chest or to a blocked or runny nose? Chest only ………….[1]*
6. *Nose only………….[2]*
7. *Both …………………[3*]

Mothers or caregivers of children under five who answered “*yes*” to the first question were selected and were eligible to answer the second question. Likewise, if their response to the second question was “*yes*,” they were further selected and eligible to answer the third question. Therefore, the outcome variable (ARI) was categorized into three, namely: chest only, nose only, and both chest and nose.

### 2.5 Predictor variables

We considered maternal, household, and child-related factors in this study. The rationale for choosing these factors at different levels is that they may influence the ARI among children under age five differently.

#### 2.5.1 Maternal factors

Maternal-related factors comprised the age of the mother (15–29, 30–39, 40–49), educational level of the mother (no education, primary, secondary/higher), mother's place of residence (urban, rural), and mother's ecological zone of residence (coastal zone, middle belt, northern zone). Others are, religious affiliation of mother (Orthodox, Pentecostal/Charismatic other Christian, Islam, Traditional/Spiritualist, no religion), literacy level of mother (illiterate, semi-literate, literate), ethnicity of mother (Akan, Ga/Dangme, Ewe, Mole-Dagbani, Gurma, Mande), and marital status of mother (not married, currently married, cohabiting, and formerly married).

#### 2.5.2 Household factors

We considered the following household-level factors in the study: sex of household head (male, female), age of household head (20–29, 30–39, 40–49, 50–59, 60–69, 70+), and household wealth quintile (poorest, poorer, middle, richer, richest). Additional variables considered were the household's drinking water source, type of toilet facility, and type of cooking fuel used. The classification of “*household source of drinking water” and the type of toilet facility* followed the WHO/United Nations International Children's Emergency Fund Joint Monitoring Programme for Water Supply, Sanitation and Hygiene (WHO/UNICEF-JMP) classification. In this study, drinking water sources were categorized as either improved or unimproved. Improved sources included pipe-borne water inside the dwelling piped into the dwelling, pipe to yard/plot, piped to the neighbor's house/compound, tube well water, borehole, protected dug well, protected, protected spring and rainwater collection, bottled water, and sachet water. Unimproved sources encompassed unprotected wells, springs, unprotected springs, rivers/dams, tanker trucks, and carts with small tanks. Toilet facilities were also divided into improved or unimproved types. Improved facilities comprised flushing to pipe sewers, flushing to septic tanks, flushing to pit latrines, flushing to unknown places, flushing to biodigesters, ventilated improved pit latrines (VIPs), pit latrines with slabs, pit toilet latrines, and composting toilets. Unimproved facilities included flush to somewhere else, a pit without slab/open pit, no facility, bush/field, and hanging toilet/latrine. The type of household cooking fuel was categorized as liquefied petroleum gas (LPG), charcoal, fuel wood, and other cooking fuel (straw/shrub/grass, crops, and animal dung). Earlier studies have applied similar measurements and categorization of these factors (16, 17).

#### 2.5.3 Child factors

The child-related factors considered in the study were the sex of the child (boy, girl), the current age of the child (<12 months, 12 months, 24 months, 36 months, 48 months), child anemia status (aneamic, not anemic) and child currently breastfeeding (no, yes). Other child-related factors include episode of fever in the last 2 weeks (no, yes), episode of diarrhea in the last 2 weeks (no, yes), birth size or weight (very large, larger than average, average, smaller than average, very small), vaccination history (never vaccinated, ever vaccinated), vitamin uptake (no, yes), and complementary food intake (no, yes).

### 2.6 Statistical analysis

The analyses of the data were performed in three stages using SPSS version 27. The first stage was the use of simple descriptive statistics to describe the outcome and predictor variables. The second stage involved a bivariate analysis or cross-tabulation of all the maternal, household, and child-related factors against children with ARI aged 6–59 months. In the third stage, we developed two multinomial regression models to examine the effect of maternal, household, and child-level factors on ARI among children aged 6–59 months. A multinomial regression model was deemed appropriate for this analysis for the following reasons. First, the dependent variable (ARI) consisted of more than two unordered categories (chest problems only, runny nose only, and both chest and nose problems). Second, this model allows for the estimation of the probability of each possible outcome of the dependent variable, given the values of the independent variables. Lastly, it enables an assessment of how changes in predictor variables influence the odds of being in a specific outcome category relative to a reference category. Prior to conducting the regression analysis, preliminary checks were preformed to assess key assumptions, including normality, linearity, multi collinearity, and homogeneity of variance. Multicollinearity was tested by using the Variance Inflation Factor (VIF) and Pearson correlation coefficients. Variables with a VIF of five or higher were excluded from the regression models to avoid multicollinearity issues. In addition, in cases where two variables were highly correlated (correlation coefficient ≥ 0.7), only one of the variables was retained for inclusion in the final model.

Model I analyzed the effect of maternal, household, and child-related factors on nose breathing difficulty only among children with ARI, and model II analyzed maternal, household, and child-related factors predicting both nose and chest breathing difficulty among children with ARI in Ghana. All variables were considered statistically significant at the 95% confidence interval (*p* < 0.05).

## 3 Results

### 3.1 Acute respiratory infections among children aged 6–59 months in Ghana

**Figure 1:**
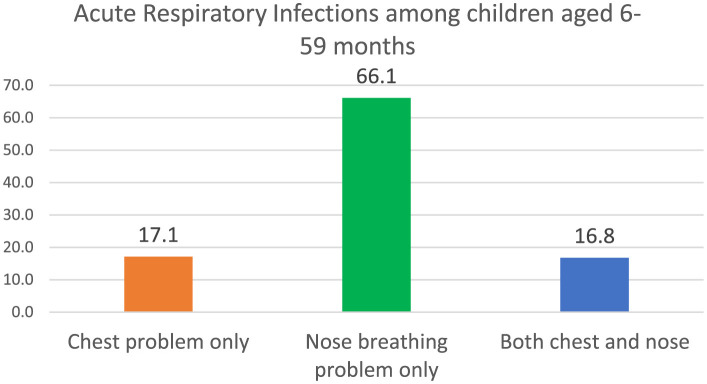
Acute respiratory infections among children aged 6–59 months. Source: Computed from the 2022 Ghana Demographic and Health Survey (GDHS).

Figure 1 shows that out of the 541 children with ARI, 17.1% had chest problems only, and 66.1% and 16.8% had nose breathing problems only and both chest and nose breathing problems, respectively.

### 3.2 Description of predictor variables in the study

Table 1 shows the percentage distribution of maternal, household, and child-related factors considered in this study. The highest proportions (44.3%) of mothers with children experiencing ARI are between the ages of 15–29 years. Approximately five out of 10 mothers of children experiencing ARI had attained a secondary or higher education level. Most (59.1%) mothers whose children have ARI reside in rural areas, and ~38% of them dwell in the Northern zone of Ghana. A greater proportion (36.9%) belonged to the Pentecostal/Charismatic faith compared to other religious affiliations, while more than half (56.6%) of them were illiterate. Most (32.2%) of them belong to the Akan ethnic group with close to two-thirds currently married.

**Table 1:** Distribution of maternal, household, and child-related factors.

**Maternal related factors**	**Weighted sample *n* = 541**	**%**
**Age of mother**		
15–29	240	44.3
30–39	230	42.4
40–49	72	13.2
**Educational level of mother**		
No education	159	29.3
Primary	92	17.0
Secondary+	291	53.7
**Place of residence of mother**		
Urban	221	40.9
Rural	320	59.1
**Ecological zone of residence of mother**		
Coastal zone	186	34.3
Middle belt	151	27.9
Northern zone	204	37.7
**Religious affiliation of mother**		
Orthodox	90	16.6
Pentecostal/Charismatic	200	36.9
Other Christian	77	14.2
Islam	141	26.1
Traditional/Spiritualist	19	3.4
No religion	15	2.8
**Literacy level of mothers**		
Illiterate	306	56.6
Semi-literate	53	9.9
Literate	182	33.5
**Ethnicity of mothers**		
Akan	174	32.2
Ga/Dangme	23	4.2
Ewe	93	17.2
Mole-Dagbani	136	25.0
Gurma	85	15.7
Mande	30	5.6
**Current marital status of mothers**		
Never married	50	9.2
Currently married	338	62.5
Cohabiting	123	22.7
Formerly married	31	5.6
**Household related factors**		
**Sex of household head**		
Male	397	73.4
Female	144	26.6
**Age of household head**		
20–29	81	14.9
30–39	196	36.2
40–49	135	24.9
50–59	60	11.2
60–69	51	9.4
70+	19	3.5
**Household source of drinking water**		
Improved water source	447	82.5
Unimproved water source	95	17.5
**Household type of toilet facility**		
Improved toilet	267	49.4
Unimproved toilet	274	50.6
**Household type of cooking fuel**		
Liquified petroleum gas (LPG)	75	13.9
Charcoal	140	25.9
Wood	326	60.2
**Household wealth index**		
Poorest	152	28.0
Poorer	119	22.0
Middle	116	21.5
Richer	101	18.7
Richest	53	9.9
**Child related factors**		
**Sex of child**		
Boy	294	54.3
Girl	248	45.7
**Current age of child**		
< 12 months	126	23.2
12 months	154	28.5
24 months	99	18.4
36 months	75	13.8
48 months	88	16.2
**Child anemia status**		
Anemic	147	27.1
Not anemic	395	72.9
**Child currently breastfeeding**		
No	249	45.9
Yes	293	54.1
**Child had fever in the last 2 weeks**		
No	300	55.3
Yes	242	44.7
**Child had diarrhea in the last 2 weeks**		
No	383	70.7
Yes	159	29.3
**Child birth size or weight**		
Very large	59	10.8
Larger than average	120	22.1
Average	312	57.7
Smaller than average	31	5.8
Very small	19	3.6
**Child vaccination history**		
Never vaccinated	7	1.3
Ever vaccinated	534	98.7
**Child vitamin uptake**		
No	69	12.8
Yes	472	87.2
**Child complementary food intake**		
No	463	85.6
Yes	78	14.4

Source: Computed from the 2022 Ghana Demographic and Health Survey (GDHS).

% = percent.

Concerning household-related factors, ~73% of children with ARI belonged to male-headed households. The highest proportions (36.2%) of heads of household were between the ages of 30 and 39 years. Approximately eight out of 10 children with ARI belong to households that access improved sources of drinking water, while 50.6% of them belong to households that access unimproved toilet facilities. Additionally, 60.2% of children with ARI belong to households that use fuelwood as a main type of cooking fuel. The highest proportion (28%) belongs to the poorest household wealth index category.

Regarding child-related factors, among children with ARI, a little more than half (54.3%) were boys. The highest proportions, ~29%, were 24 months old. Around 70% of the children with ARI were not anemic. Additionally, more than half (54.1%) were being breastfed at the time of the survey. Regarding recent health symptoms, ~45% of children with ARI had experienced fever in the 2 weeks preceding the survey, while about 29% had suffered from diarrhea during the same period. In terms of birth characteristics, over half (57.7%) were reported to have an average birth size. A significant majority (98.7%) had received at least one vaccination. Furthermore, about eight out of 10 children with ARI symptoms were reported to have received vitamin supplements, while majority (85.6%) had not been introduced to any form of complementary foods at the time of the survey.

### 3.3 Association between maternal, household, and child-related factors and ARI among children aged 6–59 months in Ghana

Table 2 shows the strength of association with chi-square analyses between maternal, household and, child-related factors and ARI among children aged 6–59 months in Ghana. Maternal-related factors, including the age, (*p* < 0.001) educational level of the mother (*p* < 0.01), place of residence of the mother (*p* < 0.05), religious affiliation of the mother (*p* < 0.05), and literacy level of the mother (*p* < 0.05), mothers' ethnicity (*p* < 0.01) was found to be significantly associated with ARI among children at *p* < 0.05. Regarding household-related factors, the age of household heads (*p* < 0.01) household source of drinking water (*p* < 0.05), household type of toilet facility (*p* < 0.01), and household wealth index (*p* < 0.001) were significantly associated with ARI among children aged 6–59 months in Ghana at *p* < 0.05. With child-related factors and ARI among children aged 6–59 months in Ghana. A significant association was established between sex of child (*p* < 0.01), current age of child (*p* < 0.01), children who experienced fever (*p* < 0.001), and experience of ARI among children under age five in Ghana.

**Table 2:** Association between maternal, household, and child-related factors and ARI among children aged 6–59 months.

**Factors**	**Acute respiratory infections among children under 5 years in Ghana**			
	**Chest only**	**Nose only**	**Both**	***P*** **-values**
**Maternal related factors**				
**Age of mother**				
15–29	11.3	72.9	15.8	0.000^***^
30–39	17.4	65.7	17.0	
40–49	35.2	45.1	19.7	
**Educational level of mother**				
No education	23.9	56.0	20.1	0.008^**^
Primary	16.5	63.7	19.8	
Secondary+	13.7	72.5	13.7	
**Place of residence of mother**				
Urban	13.1	71.9	14.9	0.043^*^
Rural	19.9	62.0	18.1	
**Ecological zone of residence of mother**				
Coastal zone	16.1	65.1	18.8	0.825
Middle belt	17.9	68.2	13.9	
Northern zone	17.2	65.7	17.2	
**Religion of mother**				
Orthodox	22.2	67.8	10.0	0.019^*^
Pentecostal/charismatic	17.5	68.0	14.5	
Other christians	13.0	66.2	20.8	
Islam	12.7	67.6	19.7	
Traditional/spiritualist	35.0	30.0	35.0	
No religion	28.6	57.1	14.3	
**Literacy level of mother**				
Illiterate	21.6	60.5	18.0	0.013^*^
Semi-literate	15.1	69.8	15.1	
Literate	10.4	74.7	14.8	
**Ethnicity of mothers**				
Akan	22.9	65.7	11.4	0.001^**^
Ga/Dangme	4.3	60.9	34.8	
Ewe	18.1	67.0	14.9	
Mole-Dagbani	11.0	75.0	14.0	
Gurma	17.6	52.9	29.4	
Mande	16.1	64.5	19.4	
**Marital status of mothers**				
Never married	14.3	75.5	10.2	0.168
Currently married	15.4	65.1	19.5	
Cohabiting	23.0	63.9	13.1	
Formerly married	16.1	74.2	9.7	
**Household related factors**				
**Sex of household head**				
Male	16.9	65.5	17.6	0.682
Female	17.9	67.6	14.5	
**Age of household head**				
20–29	7.4	72.8	19.8	0.002^**^
30–39	13.3	67.2	19.5	
40–49	25.4	57.5	17.2	
50–59	21.7	63.3	15.0	
60–69	11.8	82.4	5.9	
70+	36.8	57.9	5.3	
**Source of drinking water**				
Improved	17.2	67.8	15.0	0.045^*^
Unimproved	16.0	58.5	25.5	
**Type of toilet facility**				
Improved	18.7	69.7	11.6	0.006^**^
Unimproved	15.7	62.4	21.9	
**Type of cooking fuel**				
Liquefied petroleum gas	13.3	66.7	20.0	0.453
Charcoal	19.3	68.6	12.1	
Fuel wood	17.2	65.0	17.8	
**Household wealth index**				
Poorest	17.2	57.6	25.2	0.000^***^
Poorer	27.7	58.0	14.3	
Middle	6.0	83.6	10.3	
Richer	17.8	68.3	13.9	
Richest	15.1	67.9	17.0	
**Child related factors**				
**Sex of child**				
Boy	13.6	65.0	21.4	0.001^**^
Girl	21.4	67.3	11.3	
**Current age of child**				
< 12 months	7.2	80.0	12.8	0.004^**^
12 months	14.3	66.9	18.8	
24 months	21.2	58.6	20.2	
36 months	24.0	58.7	17.3	
48 months	25.3	60.9	13.8	
**Child anemia status**				
Anemic	17.8	63.7	18.5	0.754
Not anemic	16.8	67.0	16.2	
**Child currently breastfeeding**				
No	20.6	62.9	16.5	0.156
Yes	14.3	68.6	17.1	
**Child had fever in the last 2 weeks**				
No	15.3	73.0	11.7	0.000^***^
Yes	19.4	57.4	23.1	
**Child had diarrhea in the last 2 weeks**				
No	15.2	68.8	16.0	0.105
Yes	21.4	59.7	18.9	
**Child birth size or weight**				
Very large	10.2	69.5	20.3	0.647
Larger than average	17.5	61.7	20.8	
Average	18.6	67.0	14.4	
Smaller than average	15.6	65.6	18.8	
Very small	10.5	73.7	15.8	
**Child vaccination history**				
Never vaccinated	14.3	71.4	14.3	0.955
Ever vaccinated	17.2	66.0	16.8	
**Child vitamin uptake**				
No	14.5	75.4	10.1	0.183
Yes	17.4	64.8	17.8	
**Child complementary food intake**				
No	17.1	66.7	16.2	0.638
Yes	16.7	62.8	20.5	

Source: Computed from the 2022 Ghana Demographic and Health Survey (GDHS).

^*^P < 0.05, ^**^P < 0.01, ^***^P < 0.001.

### 3.4 Maternal, household and child predictors of acute respiratory infections among children aged 6–59 months in Ghana

Table 3 shows the results of the multinomial logistics regression of maternal, household, and child predictors of ARI among children aged 6–59 months in Ghana. There is a negative statistical relationship between mother's ecological zone of residence and ARI among children aged 6–59 months. Mothers who dwell in Coastal zones (aOR = 7.89; CI: 2.26–27.60) had a higher probability of their children having ARI compared to those living in the Northern zone. Interestingly, a lower probability of developing ARI was found among children whose mothers are illiterates (aOR = 0.28; CI: 0.08–0.98) and semi-literates (aOR = 0.16; CI: 0.03–0.74) relative to those who are literates.

**Table 3:** Multinomial logistics regression of maternal, household, and child predictors of ARI among children aged 6–59 months in Ghana.

**Factors**	**Acute respiratory infections among children under five**	
**Maternal factors**	**Nose breathing difficulty only**	**Both nose and chest breathing difficulty**
	**Exp** β **[95% C.I]**	**Exp** β **[95% C.I]**
**Age of mother**		
15–29	2.15 [0.78–5.94]	0.92 [0.25–3.33]
30–39	1.76 [0.73–4.28]	0.94 [0.31–2.89]
40–49 (RC)	1.00	1.00
**Educational level of mother**		
No education	1.22 [0.45–3.29]	1.91 [0.52–7.01]
Primary	1.27 [0.47–3.45]	2.75 [0.77–9.81]
Secondary+(RC)	1.00	1.00
**Place of residence of mother**		
Urban	1.77 [0.81–3.88]	2.46 [0.89–6.78]
Rural (RC)	1.00	1.00
**Ecological zone of mother**		
Coastal zone	2.37 [0.90–6.19]	**7.89** ^**^ **[2.26–27.60]**
Middle belt	1.31 [0.52–3.31]	2.42 [0.78–7. 54]
Northern zone (RC)	1.00	1.00
**Religion of mother**		
Orthodox	0.71 [0.14–3.59]	0.61 [0.07–5.56]
Pentecostal/Charismatic	1.28 [0.26–6.23]	1.22 [0.15–10.03]
Other Christian	2.35 [0.39–14.19]	4.76 [0.48–47.24]
Islam	1.50 [0.31–7.37]	3.45 [0.41–29.34]
Traditionalist/Spiritualist	0.27 [0.04–2.08]	1.67 [0.14–20.27]
No religion (RC)	1.00	1.00
**Literacy level of mothers**		
Illiterate	**0. 34** ^*^ **[0.12–0.93]**	**0.16** ^**^ **[0.04–0.60]**
Semi-literate	0.32 [0.10–1.08]	**0.16** ^*^ **[0.03–0.74]**
Literate (RC)	1.00	1.00
**Ethnicity of mothers**		
Akan	**0.19** ^*^ **[0.04–0.87]**	**0.12** ^*^ **[0.02–0.85]**
Ga/Dangme	0.66 [0.05–8.73]	1.02 [0.06–18.19]
Ewe	0.24 [0.05–1.23]	0.13 [0.02–1.00]
Mole-Dagbani	1.03 [0.23–4.54]	0.75 [0.12–4.49]
Gurma	0.42 [0.09–2.03]	0.86 [0.13–5.57]
Mande (RC)	1.00	1.00
**Marital status of mothers**		
Not married	0.94 [0.17–5.29]	2.00 [0.18–22.79]
Currently married	0.79 [0.18–3.60]	1.45 [0.18–11.50]
Cohabiting	0.51 [0.12–2.30]	0.80 [0.10–6.47]
Formerly married (RC)	1.00	1.00
**Household factors**		
**Sex of household head**		
Male	1.18 [0.54–2.58]	1.21 [0.45–3.28]
Female (RC)	1.00	1.00
**Age of household head**		
20–29	**12.01** ^**^ **[2.09–68.91]**	**59.08** ^**^ **[3.04–1,150.14]**
30–39	**10.88** ^**^ **[2.29–51.71]**	**53.05** ^**^ **[3.04–926.30]**
40–49	**4.77** ^*^ **[1.05–21.73]**	**19.19** ^*^ **[1.12–328.80]**
50–59	4.48 [0.91–22.20]	**20.32** ^*^ **[1.06–388.01]**
60–69	**12.68** ^**^ **[2.31–69.58]**	11.60 [0.51–265.71]
70+ (RC)	1.00	1.00
**Source of drinking water**		
Improved	0.76 [0.33–1.74]	0.52 [0.19–1.41]
Unimproved (RC)	1.00	1.00
**Type of toilet**		
Improved	0.55 [0.26–1.17]	**0.28** ^**^ **[0.11–0.72]**
Unimproved (RC)	1.00	1.00
**Type of cooking fuel**		
Liquefied petroleum gas	0.37 [0.08–1.73]	0.86 [0.12–5.99]
Charcoal	**0.21** ^**^ **[0.07–0.61]**	**0.24** ^*^ **[0.06–0.96]**
Wood (RC)	1.00	1.00
**Wealth index**		
Poorest	0.55 [0.09–3.36]	1.00 [0.09–10.70]
Poorer	0.37 [0.07–1.97]	0.49 [0.05–4.44]
Middle	**6.04** ^*^ **[1.20–30.51]**	4.02 [0.51–31.78]
Richer	1.51 [0.40–5.68]	1.55 [0.28–8.41]
Richest (RC)	1.00	1.00
**Child related factor**		
**Sex of child**		
Boy	1.55 [0.85–2.81]	**3.44** ^**^ **[1.61–7.34]**
Girl (RC)	1.00	1.00
**Current age of child**		
< 12 months	**29.07** ^***^ **[9.21–136.11]**	**21.04** ^**^ **[3.34–132.46]**
12 months	**7.92** ^**^ **[2.34–26.78]**	**6.53** ^*^ **[1.47–28.99]**
24 months	2.57 [0.84–7.83]	3.61 [0.89–14.74]
36 months	0.91 [0.34–2.41]	1.11 [0.31–3.99]
48 months (RC)	1.00	1.00
**Child anemic status**		
Anemic	0.65 [0.34–1.27]	0.56 [0.24–1.29]
Not anemic (RC)	1.00	1.00
**Child currently breastfeeding**		
No	**2.62** ^*^ **[1.15–5.94]**	2.57 [0.96–6.90]
Yes (RC)	1.00	1.00
**Child had fever in the last 2 weeks**		
No	0.93 [0.50–1.73]	**0.33** ^**^ **[0.15–0.71]**
Yes	1.00	1.00
**Child had diarrhea in the last 2 weeks**		
No	**2.28** ^*^ **[1.19–4.38]**	1.97 [0.87–4.44]
Yes	1.00	1.00
**Child birth size or weight**		
Very large	2.07 [0.30–14.12]	1.97 [0.19–20.40]
Larger than average	0.80 [0.14–4.71]	0.65 [0.08–5.64]
Average	3.40 [0.57–20.29]	1.89 [0.21–17.13]
Smaller than average	1.10 [0.15–8.15]	1.05 [0.09–12.42]
Very small	1.00	1.00
**Child vaccination history**		
Never vaccinated	1.70 [0.11–26.49]	0.94 [0.03–29.52]
Ever vaccinated	1.00	1.00
**Child vitamin uptake**		
No	0.41 [0.13–1.25]	**0.19** ^*^ **[0.04–0.83]**
Yes	1.00	1.00
**Child complementary food intake**		
No	1.42 [0.54–3.73]	1.22 [0.39–3.78]
Yes	1.00	1.00

Source: Computed from 2022 Ghana Demographic and Health Survey (GDHS).

^*^P < 0.05, ^**^P < 0.01, ^***^P < 0.001.

aOR, Adjusted Odds Ratio.

All values in bold are significant predictors of ARI.

Additionally, mothers who belong to the Akan ethnic group had lower likelihood of their children experiencing only nose breathing difficulty (aOR = 0.19; CI: 0.04–0.87) and both nose and chest breathing difficulties (aOR = 0.12; CI: 0.02–0.85) compared to mothers who belong to the Mande ethnic group.

Regarding the age of household heads, the likelihood of having breathing difficulties through the nose were higher among children whose household heads were 20–29 years (aOR = 12.01; CI: 2.09–68.91), 30–39 years (aOR = 10.88; CI: 2.29–51.71), 40–49 years (aOR = 4.77; CI: 1.05–21.13), and 60–69 years (aOR = 12.68; CI: 2.31–69.58) than children dwelling with heads aged 70 years and above. Additionally, the probability of children having both nose and chest breathing difficulty was higher among children living with household heads aged 20–29 years (aOR = 59.08; CI: 3.04–1150.14), 30–39 years (aOR = 59.08; CI: 3.04–926.30), 40–49 years (aOR = 19.19; CI: 1.12–328.80), and 50–59 years (aOR = 20.32; CI: 1.06–388.01) compared to children living with heads aged 70 years and above.

Furthermore, children who belong to households with improved type of toilet facilities (aOR = 0.28; CI: 0.11–0.72) had lower odds of experiencing both nose and chest breathing difficulties compared to those living in households with unimproved toilet facilities. Children in households using charcoal as cooking fuel are less likely to experience difficulty in breathing through the nose only (aOR = 0.21; CI: 0.07–0.61) and both nose and chest breathing difficulties (aOR = 0.24; CI: 0.06–0.96) than children in households that use fuelwood.

Regarding child-related factors, the sex of the child significantly predicted ARI among children, thus boys were 3.44 times more likely to have both nose and chest breathing difficulties than girls. The current age of the child also significantly predicted ARI among children under age five. Children who were < 12 months old (aOR = 29.07; CI: 9.21–136.11) and 24 months old (aOR = 7.92; CI: 2.34–26.78) were more likely to have difficulty breathing through their nose compared to those who are 48 months old. Similarly, children < 12 months (aOR = 21.04; CI: 3.34–132.46) and those 12 months old (aOR = 6.53; CI: 1.47–28.99) have a high probability of experiencing both nose and chest breathing difficulties relative to children who are 48 months old. Children who were not being breastfed at the time of the survey had significantly higher odds of experiencing breathing difficulties through the nose (aOR = 2.62; CI: 1.15–5.94) compared to those who were currently being breastfed. Moreover, children who had not experienced fever (aOR = 0.33; CI: 0.15–0.17) in the 2 weeks preceding the survey were less likely to have both nasal and chest breathing difficulties than those who had experienced fever. Additionally, children who had suffered from diarrhea (aOR = 2.28; CI: 1.19–4.38) during the 2 weeks prior to the survey had increased odds of experiencing breathing difficulties through both the nose and chest compared to those who had not had diarrhea.

## 4 Discussion

Our study observed a decline in chest problems from 25.7% in 2014 to 17.1% in 2022, comparing this result with the 2014 GDHS findings, it shows that 25.7% of children had chest problems only which means that there has been an 8.6% decrease in this prevalence. Furthermore, the 2014 GDHS findings indicated that 46.4% of children had nose breathing problems, which shows that the prevalence has increased by 19.7%. Again, there has also been a decrease in the proportion of children with both chest and nose breathing problems from 27.9% in 2014 to 16.8% in 2022 in Ghana. It is also important to note that the number of children with ARI has increased from 372 in 2014 to 541 in 2022. This outcome is likely due to expanded preventive health measures, particularly vaccination programs, improvements in environmental and sanitation and improved healthcare access and utilization. Similar trends were found in studies by Brooks et al. (18) in Bangladesh and Hammitt et al. (19) in Kenya, which reported reductions in childhood pneumonia and ARIs, following the introduction of pneumococcal conjugate vaccines (PCV) and improved healthcare systems. In agreement with our findings, Fujita et al. (20) noted that access to antibiotics and healthcare advancements in countries like Ethiopia have helped lower ARI complications. Additionally, the decline in children experiencing both chest and nasal breathing problems aligns with results from Weary et al. (21) in Uganda, where integrated health programs effectively reduced respiratory infections. Conversely, the significant rise in nasal breathing problems (46.4% to 66.1%) is consistent with findings from Ohemeng et al. (22) in Ghana and Raju et al. (23) which emphasize the impact worsening air quality and indoor pollution on upper respiratory tract infections (URTIs). Similarly, Fan et al. (24) in China linked an increase in URTIs to urbanization and air pollution. Moreover, the growing number of children with ARI reflects an ongoing public health challenge, underscoring that ARIs remain a major cause of child morbidity and mortality globally due to factors like population growth, limited healthcare access, and antimicrobial resistance (25–29).

Maternal factors, such as the ecological location of residence, significantly influence the likelihood of children developing respiratory infections. Children in coastal areas were also at greater risk, possibly due to increased humidity and the presence of allergens such as mold and sea salt aerosols, which aggravate respiratory conditions. Existing studies have confirmed that these environmental factors in coastal zones contribute to higher incidences of respiratory infections (30–32). Maternal literacy also emerged as a critical determinant. Although illiteracy was paradoxically associated with lower odds of respiratory infections, this might be explained by lower health-seeking behavior or underreporting of symptoms in populations with lower education levels. Daffe et al. (32) highlight the general trend that maternal literacy is protective for child health, as educated mothers are more likely to recognize symptoms and seek medical care. However, this paradox may be attributed the potential underreporting or reporting biases of respiratory symptoms by less literate mothers or reduced access to health facilities, as suggested by Naz and Ghimire (33). Thus, the relationship between maternal literacy and respiratory infections may be complex, with cultural and reporting behaviors influencing the results. We suggest further studies and in-depth qualitative research into the relationship between mothers' literacy level and ARI infections among their children to either confirm or refute this study's findings. Moreover, ethnic differences were observed, with children from the Akan ethnic groups experiencing lower odds of respiratory difficulties. This finding may reflect cultural health practices or variations in environmental exposures, as previous studies have shown that certain ethnic groups tend to have better health outcomes due to specific cultural practices or healthcare-seeking behaviors (34–36).

The Akan ethnic group predominately resides in the southern regions of Ghana, such as Ashanti, Greater Accra Eastern and Central regions. These areas are generally more urbanized and economically developed compared to other parts of the country. Urban settings often offer better access to healthcare facilities which encourages Akan mothers to be more inclined toward biomedical practices leading to better health outcomes for their children (37, 38).

However, Ganle (39) contradicts these findings, arguing that ethnic disparities in healthcare access often put minority groups at a disadvantage, increasing their vulnerability to childhood infections. This indicates that ethnicity can interact with various social and environmental factors to influence child health outcomes in different ways.

Household factors, such as the age of the household head, were significantly associated with the likelihood of children experiencing respiratory infections. Younger household heads, particularly those between 20 and 39 years, were more likely to have children with respiratory difficulties. This could be attributed to their generally lower socioeconomic status and less experience in managing household health risks. Existing studies have affirmed that younger heads of households often have fewer resources and may live in environments with poor ventilation or substandard housing (29, 38–40). In addition, improved sanitation was found to be protective against respiratory infections in children. Households with improved toilet facilities had lower odds of respiratory difficulties, which align with existing evidence that better sanitation reduces exposure to pathogens and indoor air pollution, contributing to a healthier environment (41, 42). Furthermore, the type of cooking fuel used was an important determinant. While solid fuels like wood have long been known to increase respiratory infection risks due to indoor air pollution, this study surprisingly found that households using charcoal had lower odds of respiratory difficulties. Woolley et al. (43) reported that both wood and charcoal increase respiratory risks due to the smoke produced. However, this study's results may reflect local practices, as households using charcoal may have better ventilation or use outdoor cooking areas, which reduce children's exposure to harmful smoke. Furthermore, charcoal combustion typically produces less smoke and fewer pollutants than wood. This is because charcoal undergoes a carbonization process that removes moisture and volatile compounds, resulting in a cleaner-burning fuel. In contrast, wood combustion releases higher levels of particulate matter and other harmful pollutants, increasing the risk of respiratory issues.

Child-related factors such as boys were found to be more vulnerable to respiratory infections compared to girls. This observation is consistent with existing studies that suggest boys have a higher biological susceptibility to respiratory infections, likely due to differences in immune system function and lung development (44–46). The age of the child also significantly influenced the likelihood of respiratory difficulties. Infants under 12 months were especially prone to respiratory infections, reflecting their underdeveloped immune systems and smaller airways, which make them more susceptible to such conditions. This finding is supported by existing studies which noted that infants, particularly those under 1 year of age, are more likely to suffer from acute respiratory infections due to these physiological vulnerabilities (13, 46–48). These child-related factors highlight the importance of targeted interventions for vulnerable age groups and the need to consider gender differences in respiratory health. These findings underscore the need for designing strategies, policies, and programme to prevent ARIs among children under five through a holistic and integrated approach. Addressing maternal, household, and child-related socio-demographic factors collectively is important for develop effective interventions. Key policy actions should include investments in female literacy programs and maternal health education, the promotion and the adoption of clean cooking technologies, scaling up nutrition programmes targeting children under five and culturally sensitive health education campaigns that dispel misconceptions about ARIs.

## 5 Study limitations

The study has the following limitations. First, it relies on existing (secondary) data that is cross-sectional in nature. Consequently, it cannot be used to infer causality but only establish associations between the variables of interest. Second, the data were collected 3 years ago. Although there has been a general decline in ARI among children since then, the proximate determinants may have changed over time. Third, the occurrence of ARI among children is self-reported and not clinically verified, which may introduce social desirability bias. Additionally, the study is limited in its inclusion of some maternal, household and child related predictors of ARI among children, which may not be exhaustive due to the constraints of secondary data.

Finally, there were some very wide confidence intervals in the regression results which indicate potential issues related to sparse data, overlifting or instability in the model. On the sparse data issue, if the number of observations in a subgroup (for example household heads aged 20–29) is small, especially with few events (disease cases), the model may struggle to estimate the effect precisely. This may lead to high uncertainty, which inflates the confidence interval. Again, there is potential overfitting occurs when the model is too complex for the amount of available data such as having too many predictors or interaction terms relative to the number of events. This makes the estimates unstable and overly sensitive to small changes in the data, resulting in exaggerated odds ratios and very wide confidence intervals. However, these limitations, did not significantly affect the accuracy and robustness of the result obtained.

## 6 Conclusion

Findings from the 2022 GDHS indicate that a considerable proportion of children aged 6–59 months in Ghana experienced ARIs. Key determinants significantly associated with ARI prevalence included maternal literacy level, household sanitation, ecological location of residence, and the child's demographics, nutritional and health status. These findings highlight the need for targeted public health interventions that prioritize improving maternal literacy, improving household living conditions, and raising caregiver awareness on ARI prevention. Such focused efforts are essential to reducing the burden of ARIs among this vulnerable age group. A comprehensive, cross-sectoral response is critical to reducing ARI prevalence and improving respiratory health outcome for vulnerable children across diverse communities.

## Data Availability

The datasets presented in this study can be found in online repositories. The names of the repository/repositories and accession number(s) can be found below: https://dhsprogram.com.

## References

[1] Gebrerufael GG Hagos BT. Prevalence and predictors of acute respiratory infection among children under-five years in Tigray regional state, northern Ethiopia: a cross-sectional study. BMC Infect Dis. (2023) 23:743. 10.1186/s12879-023-08701-2 37904115 PMC10614314

[2] Roselany R Surjono E. Pneumonia clinical features in under-five children treated in Atma Jaya hospital in 2017–2020. Cough. (2023) 135:91–2. 10.15395/mkb.v55n1.2966

[3] Ekholuenetale M Nzoputam CI Okonji OC Barrow A Wegbom AI Edet CK. Differentials in the prevalence of acute respiratory infections among under-five children: an analysis of 37 sub-Saharan countries. Global Pediatric Health. (2023) 10:2333794X231156715. 10.1177/2333794X231156715 36814530 PMC9940173

[4] Ghana Statistical Service Macro Systems. Institute for Resource Development. Demographic, Health Surveys. Ghana demographic and health survey. Accra: Ghana Statistical Service (2023).

[5] Kolekang A Sarfo B Danso-Appiah A Dwomoh D Akweongo P. Contribution of child health interventions to under-five mortality decline in Ghana: a modeling study using lives saved and missed opportunity tools. PLoS ONE. (2022) 17:e0267776. 10.1371/journal.pone.0267776 35913919 PMC9342718

[6] Walker CL Rudan I Liu L Nair H Theodoratou E Bhutta ZA. Global burden of childhood pneumonia and diarrhoea. Lancet. (2013) 381:1405–16. 10.1016/S0140-6736(13)60222-6 23582727 PMC7159282

[7] Sharrow D Hug L You D Alkema L Black R Cousens S. Global, regional, and national trends in under-5 mortality between 1990 and 2019 with scenario-based projections until 2030: a systematic analysis by the UN Inter-agency group for child mortality estimation. Lancet Global Health. (2022) 10:e195–206. 10.1016/S2214-109X(21)00515-5 35063111 PMC8789561

[8] Selvaraj K Chinnakali P Majumdar A Krishnan IS. Acute respiratory infections among under-5 children in India: a situational analysis. J Nat Sci Biol Med. (2014) 5:15. 10.4103/0976-9668.127275 24678190 PMC3961922

[9] McAllister DA Liu L Shi T Chu Y Reed C Burrows J. Global, regional, and national estimates of pneumonia morbidity and mortality in children younger than 5 years between 2000 and 2015: a systematic analysis. Lancet Global Health. (2019) 7:e47–57. 10.1016/S2214-109X(18)30408-X 30497986 PMC6293057

[10] World Health Organization. Neonatal and perinatal mortality: country, regional and global estimates. World Health Organization (2006).

[11] Sonego M Pellegrin MC Becker G Lazzerini M. Risk factors for mortality from acute lower respiratory infections (ALRI) in children under five years of age in low and middle-income countries: a systematic review and meta-analysis of observational studies. PLoS ONE. (2015) 10:e0116380. 10.1371/journal.pone.0116380 25635911 PMC4312071

[12] Afari-Asiedu S Oppong FB Tostmann A Ali Abdulai M Boamah-Kaali E Gyaase S. Determinants of inappropriate antibiotics use in rural central Ghana using a mixed methods approach. Front Public Health. (2020) 8:90. 10.3389/fpubh.2020.00090 32266200 PMC7105730

[13] Mir F Ariff S Bhura M Chanar S Nathwani AA Jawwad M. Risk factors for acute respiratory infections in children between 0 and 23 months of age in a peri-urban district in Pakistan: a matched case–control study. Front Pediatr. (2022) 9:704545. 10.3389/fped.2021.704545 35083182 PMC8784846

[14] Odo DB Yang IA Dey S Hammer MS van Donkelaar A Martin RV. Ambient air pollution and acute respiratory infection in children aged under 5 years living in 35 developing countries. Environ Int. (2022) 159:107019. 10.1016/j.envint.2021.107019 34875446

[15] Makoka D. The impact of maternal education on child nutrition: evidence from Malawi, Tanzania, and Zimbabwe. ICF Int. (2013). 26297004 10.1186/s12887-015-0406-8 PMC4546212

[16] Klu D Agordoh PD. Sex of household head and other household determinants of childhood anaemia among households in Ghana: regression analysis of the 2019 malaria indicator survey. J Health Popul Nutr. (2022) 41:46. 10.1186/s41043-022-00327-5 36217188 PMC9549624

[17] Klu D Atiglo DY Christian AK. Multinomial logistic regression analysis of the determinants of anaemia severity among children aged 6–59 months in Ghana: new evidence from the 2019 Malaria Indicator Survey. BMC Pediatr. (2023) 23:91. 10.1186/s12887-023-03919-0 36850016 PMC9969679

[18] Brooks WA Zaman K Goswami D Prosperi C Endtz HP Hossain L. The etiology of childhood pneumonia in Bangladesh: findings from the Pneumonia Etiology Research for Child Health (PERCH) study. Pediatr Infect Dis J. (2021) 40:S79–90. 10.1097/INF.0000000000002648 34448747 PMC8448409

[19] Hammitt LL Etyang AO Morpeth SC Ojal J Mutuku A Mturi N. Effect of ten-valent pneumococcal conjugate vaccine on invasive pneumococcal disease and nasopharyngeal carriage in Kenya: a longitudinal surveillance study. Lancet. (2019) 393:2146–54. 10.1016/S0140-6736(18)33005-8 31000194 PMC6548991

[20] Fujita AW Werner K Jacob JT Tschopp R Mamo G Mihret A. Antimicrobial resistance through the lens of one health in Ethiopia: a review of the literature among humans, animals, and the environment. Int J Infect Dis. (2022) 119:120–9. 10.1016/j.ijid.2022.03.041 35358724 PMC9107604

[21] Weary TE Tusiime P Tuhaise S Mandujano Reyes JF Ross E Gern JE. Respiratory disease patterns in rural Western Uganda, 2019–2022. Front Pediatr. (2024) 12:1336009. 10.3389/fped.2024.1336009 38650995 PMC11033374

[22] Ohemeng A Marquis GS Lartey A. Household food insecurity is associated with respiratory infections among 6–11-Month old infants in rural ghana. Pediatr Infect Dis J. (2015) 34:821–5. 10.1097/INF.0000000000000743 25961890

[23] Raju S Siddharthan T McCormack MC. Indoor air pollution and respiratory health. Clin Chest Med. (2020) 41:825–43. 10.1016/j.ccm.2020.08.014 33153698 PMC7665158

[24] Fan Y Ding X Hang J Ge J. Characteristics of urban air pollution in different regions of China between 2015 and 2019. Build Environ. (2020) 180:107048. 10.1016/j.buildenv.2020.107048

[25] Li Y Wang X Blau DM Caballero MT Feikin DR Gill CJ. Global, regional, and national disease burden estimates of acute lower respiratory infections due to respiratory syncytial virus in children younger than 5 years in 2019: a systematic analysis. Lancet. (2022) 399:2047–64. 35598608 10.1016/S0140-6736(22)00478-0 PMC7613574

[26] Nair H Nokes DJ Gessner BD Dherani M Madhi SA Singleton RJ. Global burden of acute lower respiratory infections due to respiratory syncytial virus in young children: a systematic review and meta-analysis. Lancet. (2010) 375:1545–55. 10.1016/S0140-6736(10)60206-1 20399493 PMC2864404

[27] Swere KM. Challenges hindering the accessibility of Tanzania's health service: a literature review. Int J Econ Financ. (2016) 8:242–5. 10.5539/ijef.v8n8p24240195169

[28] Hasan AR Selim MA Anne FI Escobar-DeMarco J Ireen S Kappos K. Opportunities and challenges in delivering maternal and child nutrition services through public primary health care facilities in urban Bangladesh: a qualitative inquiry. BMC Health Serv Res. (2023) 23:1172. 10.1186/s12913-023-10094-6 37891649 PMC10612189

[29] Davey TM Cameron CM Ng SK McClure RJ. The relationship between maternal education and child health outcomes in urban Australian children in the first 12 months of life. Matern Child Health J. (2015) 19:2501–11. 10.1007/s10995-015-1771-5 26122254

[30] Laari S Takahashi S. The impact of formal maternal education on child survival in Ghana. Global J Health Sci. (2021) 6:17–33. 10.47604/gjhs.1418

[31] Mondal D Paul P. Effects of indoor pollution on acute respiratory infections among under-five children in India: evidence from a nationally representative population-based study. PLoS ONE. (2020) 15:e0237611. 10.1371/journal.pone.0237611 32797105 PMC7428171

[32] Daffe ML Thiam S Bah F Ndong A Cabral M Diop C. Household level of air pollution and its impact on the occurrence of acute respiratory illness among children under five: secondary analysis of demographic and health survey in West Africa. BMC Public Health. (2022) 22:2327. 10.1186/s12889-022-14611-w 36510195 PMC9746013

[33] Naz L Ghimire U. Unimproved water, sanitation, and hygiene (WASH) and common childhood illness in Myanmar: evidence from a nationally representative survey. (2020) *Europepmc*. 10.21203/rs.3.rs-36037/v1

[34] Demissie BW Amele EA Yitayew YA Yalew ZM. Acute lower respiratory tract infections and associated factors among under-five children visiting Wolaita Sodo University Teaching and Referral Hospital, Wolaita Sodo, Ethiopia. BMC Pediatr. (2021) 21:1–8. 10.1186/s12887-021-02888-6 34544420 PMC8451097

[35] Anaemias WN. Tools for Effective Prevention and Control. World Health Organization: Geneva, Switzerland. (2017). p. 1–83.

[36] Hasan MM Saha KK Yunus RM Alam K. Prevalence of acute respiratory infections among children in India: regional inequalities and risk factors. Matern Child Health J. (2022) 26:1594–602. 10.1007/s10995-022-03424-3 35435580 PMC9174316

[37] Denno DM Bentsi-Enchill A Mock CN Adelson JW. Maternal knowledge, attitude and practices regarding childhood acute respiratory infections in Kumasi, Ghana. Ann Trop Paediatr. (1994) 14:293–301. 10.1080/02724936.1994.11747732 7880091

[38] Seidu AA Ameyaw EK Ahinkorah BO Baatiema L Appiah F. Ecological zone and symptoms of acute respiratory infection among children under five in Ghana: 1993–2014. SSM Popul Health. (2019) 8:100414. 10.1016/j.ssmph.2019.100414 31206003 PMC6558297

[39] Ganle JK. Ethnic disparities in utilisation of maternal health care services in Ghana: evidence from the 2007 Ghana Maternal Health Survey. Ethn Health. (2016) 21:85–101. 10.1080/13557858.2015.1015499 25728254

[40] Vousden N Bunch K Kenyon S Kurinczuk JJ Knight M. Impact of maternal risk factors on ethnic disparities in maternal mortality: a national population-based cohort study. Lancet Reg Health Eur. (2024) 40:100893. 10.1016/j.lanepe.2024.100893 38585675 PMC10998184

[41] Kota K Chomienne MH Geneau R Yaya S. Socio-economic and cultural factors associated with the utilization of maternal healthcare services in Togo: a cross-sectional study. Reprod Health. (2023) 20:109. 10.1186/s12978-023-01644-6 37488593 PMC10367352

[42] Gaffan N Kpozèhouen A Dégbey C Glèlè Ahanhanzo Y Glèlè Kakaï R Salamon R. Household access to basic drinking water, sanitation and hygiene facilities: secondary analysis of data from the demographic and health survey V, 2017–2018. BMC Public Health. (2022) 22:1345. 10.1186/s12889-022-13665-0 35836162 PMC9284778

[43] Woolley KE Bartington SE Kabera T Lao XQ Pope FD Greenfield SM. Comparison of respiratory health impacts associated with wood and charcoal biomass fuels: a population-based analysis of 475,000 children from 30 Low-and middle-income countries. Int J Environ Res Public Health. (2021) 18:9305. 10.3390/ijerph18179305 34501907 PMC8431364

[44] Tran HM Tsai FJ Lee YL Chang JH Chang LT Chang TY. The impact of air pollution on respiratory diseases in an era of climate change: a review of the current evidence. Sci Total Environ. (2023) 15:166340. 10.1016/j.scitotenv.2023.166340 37591374

[45] Gaydosh L. Childhood risk of parental absence in Tanzania. Demography. (2015) 52:1121–46. 10.1007/s13524-015-0411-4 26220661 PMC5434424

[46] Rahman A Hossain MM. Prevalence and determinants of fever, ARI and diarrhea among children aged 6–59 months in Bangladesh. BMC Pediatr. (2022) 22:117. 10.1186/s12887-022-03166-9 35248016 PMC8897933

[47] Troeger C Blacker B Khalil IA Rao PC Cao J Zimsen SR. Estimates of the global, regional, and national morbidity, mortality, and aetiologies of lower respiratory infections in 195 countries, 1990–2016: a systematic analysis for the Global Burden of Disease Study 2016. Lancet Infect Dis. (2018) 18:1191–210. 30243584 10.1016/S1473-3099(18)30310-4 PMC6202443

[48] Dias SP Brouwer MC van de Beek D. Sex and gender differences in bacterial infections. Infect Immun. (2022) 90:e00283–22. 10.1128/iai.00283-22 36121220 PMC9584217

